# Enhancing anesthetic techniques for improving whisker stimulation response in the barrel cortex

**DOI:** 10.1371/journal.pone.0318306

**Published:** 2025-02-25

**Authors:** Ye Yuan, Tian Liu, Jue Wang

**Affiliations:** Key Laboratory of Biomedical Information Engineering of Ministry of Education, Institute of Biomedical Engineering, School of Life Science and Technology, Xi’an Jiaotong University, Xi’an, China; University of Illinois at Urbana-Champaign, UNITED STATES OF AMERICA

## Abstract

This study adopts and validates an anesthetic protocol designed for rat whisker stimulation experiments, achieving significant enhancements in the neural response of the barrel field cortex. By combining alpha-chloralose, low-dose Isoflurane (0.5%) and Dexdomitor, the protocol not only maintains a stable anesthetic state but also markedly improves the amplitude and latency of local field potential (LFP) signals. Experimental results reveal that LFP amplitudes in the barrel field under this protocol are twice as high as those achieved with Isoflurane and four times as high as those with Ketamine-Xylazine, with significantly shortened latencies and reduced noise interference. For the first time, power spectral analysis reveals a distinct enhancement of oscillatory power in the alpha (8–13 Hz) and beta (13–30 Hz) bands under alpha-chloralose anesthesia, diverging from the traditional dominance of delta (0.5–4 Hz) oscillations observed with other anesthetics. Mechanistically, this phenomenon may be attributed to alpha-chloralose’s unique modulation of GABAergic and glutamatergic pathways, promoting cortical desynchronization and enhanced sensory processing. This protocol offers new insights into optimizing sensory-evoked neural signal acquisition and provides a reference for future studies exploring neural modulation in sensory neuroscience.

## 1. Introduction

In neurophysiological research, the barrel cortex of rats serves as a core model for processing tactile information, playing a particularly significant role in whisker stimulation experiments [1,2]. The precise anatomical structure and functional characteristics of the barrel cortex make it an ideal platform for investigating tactile perception, sensorimotor integration, and cortical neural network dynamics [3,4]. However, obtaining high-quality local field potential (LFP) signals remains a major challenge due to muscle noise interference and physiological fluctuations induced by anesthesia [5,6]. These issues not only compromise the stability and reliability of experimental data but may also obscure deeper insights into the mechanisms of sensory neural activity. Therefore, developing optimized anesthetic protocols tailored for long-term neural recordings is crucial [7,8].

Commonly used anesthetics include Isoflurane [9,10] and Ketamine-Xylazine [11,12]. While these anesthetics meet the requirements of short-term experiments, their limitations in long-term neural recordings are apparent. Isoflurane requires continuous administration to maintain its anesthetic effect, but it often causes physiological fluctuations, such as a decrease in body temperature and unstable heart rate, which can significantly impact the stability of neural signals [13,14]. In contrast, Ketamine-Xylazine provides effective sedation but has a short duration of action (approximately 30 minutes), necessitating frequent re-dosing. This increases the complexity of experimental operations and often leads to significant physiological fluctuations, reducing the reliability of experimental results [15–17]. These limitations highlight the urgent need for a stable anesthetic protocol capable of minimizing interference during long-term experiments to meet the demand for high-quality data in sensory neuroscience research [18,19].

To address these issues, some studies have explored improved anesthetic protocols. For example, Yuan et al. found that alpha-chloralose could effectively enhance the quality of raw brain signals by comparing different anesthetic protocols [20]. While this approach has demonstrated significant advantages in reducing muscle noise interference and improving signal clarity, its suitability for long-term experiments, its effect on specific LFP frequency bands, and its regulatory mechanisms on neural signals still require further investigation [21]. Building on this, the present study proposes an anesthetic protocol designed for whisker stimulation experiments. By combining alpha-chloralose with low-dose Isoflurane (0.5%) and supplementing it with Dexdomitor to suppress muscle activity, this protocol significantly improves LFP signal clarity while effectively maintaining the core physiological stability of the animals [20]. Experiments show that, compared to traditional anesthetic protocols using Isoflurane or Ketamine-Xylazine alone, this combined protocol demonstrates superior stability and reliability over the course of 7-hour experiments [22]. Moreover, Dexdomitor exhibits unique advantages in reducing noise interference caused by muscle activity, providing additional support for the accuracy of data in long-term neural recording experiments [16].

In addition to validating the practical utility of the anesthetic protocol, this study systematically investigates the frequency-domain characteristics of LFP signals under different anesthetic protocols. Power spectral analysis reveals that alpha-chloralose anesthesia significantly enhances oscillatory power in the alpha (8–13 Hz) and beta (13–30 Hz) frequency bands, in stark contrast to the delta-band-dominated characteristics commonly observed with other anesthetics [23]. This phenomenon may be attributed to alpha-chloralose’s ability to regulate GABAergic and glutamatergic pathways, promoting cortical desynchronization and enhancing neural responses. The main contributions of this paper are as follows:

1) Long-lasting and stable anesthetic protocol to enhance neural signal acquisition: This study adopts an anesthetic protocol specifically designed for whisker stimulation experiments. The protocol combines alpha-chloralose with a low concentration of Isoflurane (0.5%) and incorporates Dexdomitor to effectively suppress muscle activity. Compared to traditional anesthetic protocols, such as using Isoflurane or Ketamine-Xylazine alone, this combination not only delivers higher-quality LFP signals in the barrel cortex during whisker stimulation but also maintains stable anesthesia for up to 7 hours. More importantly, this protocol ensures long-term stability of key physiological parameters, including body temperature and heart rate, thereby mitigating the physiological fluctuations commonly observed in prolonged experiments.
2) Significant improvement in LFP signal performance and shortened response latency: Experimental results confirm that this protocol significantly outperforms conventional methods. Under alpha-chloralose anesthesia, the amplitude of barrel cortex LFP signals is approximately twice that of Isoflurane and four times that of Ketamine-Xylazine, demonstrating markedly enhanced neural responsiveness during whisker stimulation experiments. Furthermore, the protocol achieves the shortest peak latency of LFP signals, reflecting a faster and more efficient cortical response.
3) Mechanistic insights into anesthetic-induced neural activity regulation: This study systematically explores the underlying mechanisms of differences in neural signal characteristics across anesthetic protocols. Alpha-chloralose modulates GABAergic and glutamatergic pathways, achieving a unique balance between neuronal excitation and inhibition, promoting cortical desynchronization, and enhancing sensory-induced alpha and beta oscillations. In contrast, Isoflurane excessively suppresses cortical activity, while Ketamine-Xylazine’s disruption of physiological stability limits its efficacy. These mechanistic insights not only deepen our understanding of anesthetic effects on neural activity but also underscore the unique advantages of the proposed protocol in sensory neuroscience research.

The rest of this paper is organized as following: Section II states experimental setups. Section III introduces three different anesthetics methods and experimental system. The experiment results are present to validate our techniques’ superiority in Section IV. Section V concludes this paper.

## 2. Experimental setups

This section first introduces the source of animals used in our experiments. Then, we show the details of the preparing things for the experimental setups.

### 2.1. Animal source

The 30 male Sprague Dawley (SD) rats (31-12-002-D-000010), weighing 250–350 g, were purchased from the Institute of Medical Experimental Animals at the Chinese Academy of Medical Sciences. They were housed under a strict 12-hour light-dark cycle in animal room accredited by the Animal Protection and Use Committee of Xi’an Jiaotong University. All procedures were performed in accordance with protocols approved by the committee. Animals had ad libitum access to food and water, and all efforts were made to minimize suffering and ensure ethical treatment, in alignment with ARRIVE guidelines (PLoS Biol 8(6): e1000412, 2010). In this study, we conducted comparative experiments on 3 groups (G_1_ = 10, G_2_ = 10, G_3_ = 10) to evaluate the effects of three anesthesia methods: alpha-chloralose, Isoflurane, and Ketamine-Xylazine. On the experimental day, animals were randomly assigned to different anesthesia protocol groups to minimize potential biases.

### 2.2. Preparing steps for whisker stimulation experiment

To accurately collect the electroencephalogram (EEG) response signals of rats’ whisker stimulation, we followed a series of preparatory steps as outlined in [24].

1) Prepare the rats for anesthesia: Before anesthesia, the rats are placed in a 30 cm ×  10 cm ×  10 cm anesthesia chamber to acclimate. Induction anesthesia is then initiated, setting the Isoflurane concentration to 5% and the oxygen flow rate to 1.2 L/min, maintaining this for approximately 4 minutes until the rat is fully anesthetized. Throughout the induction process, it is essential to closely monitor the rat’s breathing pattern and anesthesia depth, using regular toe pinch tests to assess anesthesia levels and ensure the rat is adequately sedated without being overly anesthetized.
2) Surgical procedure: Once the animal has reached a deep level of anesthesia, it is placed on the surgical table, with its head secured to begin the procedure. During the surgery, the Isoflurane concentration was 2% and the oxygen flow rate was 1.2 L/min. After carefully removing the muscle tissue on the brain surface with a scalpel, a 1.5 mm ×  1.5 mm section of the skull on the left side of the brain is removed using a drill to expose the dura mater. The dura mater remained intact throughout the procedure to preserve physiological conditions and ensure reliable neural recordings. Dexdomitor (0.5 mg/kg i.p.) is administered via intraperitoneal injection to further relax the animal’s muscles, keeping it calm and less responsive to external stimuli. This step helps reduce the animal’s stress response, ensuring a smoother experimental process. Additionally, Dexdomitor’s sedative effect maintains a stable physiological state, minimizing potential movement interference and thereby enhancing the precision of the experimental procedures and the reliability of the data. We also use dexamethasone (0.5 mg/kg i.p.) to reduce potential inflammatory reactions and maintain the physiological stability of the animals, particularly during long-term anesthesia, ensuring the stability of electrophysiological signals and the reliability of the experimental data.
3) Recording EEG signals: We precisely positioned a 100 µm AgCl electrode with an impedance of 200 kΩ at a specified location in the left hemisphere of the rat’s brain, specifically 2.5 mm posterior and 5.5 mm lateral to the bregma, to ensure accurate signal acquisition. The ground electrode was fixed on the opposite (right) side of the skull. Subsequently, the electrodes were connected to the input interface of a Cerbus device produced by Blackrock Microsystems in Salt Lake City, USA, allowing for real-time recording and monitoring of the rat’s EEG signals to obtain high-quality data. Throughout the experiment, the animal’s vital signs, including heart rate and body temperature, were continuously monitored to maintain physiological stability. Close observation not only ensured the continuity and reliability of data collection but also met ethical standards for animal welfare, minimizing potential discomfort and stress responses. After finishing the experiments, animals were euthanized by intraperitoneal injection of pentobarbital (180 mg/kg, i.p).

## 3. Anesthetics techniques and experimental system

In this section, we first introduce three anesthetic methods (alpha-chloralose, Isoflurane, and Ketamine-Xylazine) used in animal experiments. Then we present the details of whisker stimulation experimental system.

### 3.1. Anesthetic methods

Alpha-chloralose induces a sedative state by enhancing γ-aminobutyric acid (GABA) receptor activity in rodents. Typically administered via intravenous or intraperitoneal injection for anesthesia in rats and mice, it effectively maintains the animals in a sedated or unconscious state, thereby minimizing discomfort and stress responses triggered by external stimuli. In this study, the time interval from Isoflurane induction to the administration of alpha-chloralose was approximately 25 minutes (4 minutes for induction, 15-20 minutes for surgical procedures and electrode placement, and 2-3 minutes for physiological observation). Subsequently, alpha-chloralose was administered to the rats via intraperitoneal injection at a dose of 4.0 mg/kg (i.p.). Finally, LFP signals were recorded.

Isoflurane is typically administered via inhalation, with its primary mechanism being the enhancement of γ-aminobutyric acid (GABA) receptor activity in the central nervous system and the inhibition of inhibitory neurotransmitter receptors, thereby achieving an anesthetic effect. Due to its rapid onset and favorable recovery profile, Isoflurane is widely used in animal experiments. In this experiment, to prevent SD rats from unexpectedly waking during stimulation, Isoflurane should be maintained at an anesthesia level of 1.5% to collect LFP signals from the barrel cortex following whisker stimulation.

The Ketamine-Xylazine is known for its rapid onset and short duration of anesthesia, making it an ideal choice for procedures requiring quick recovery. It is widely used in minor surgeries and diagnostic imaging to enhance anesthetic and sedative effects. Generally speaking, the anesthetic effect of Ketamine-Xylazine in rats lasts for only 30 minutes. Similarly, the time interval from Isoflurane induction to Ketamine-Xylazine administration was also approximately 25 minutes. Then, the rats were administered Ketamine (80 mg/kg) and Xylazine (10 mg/kg) via intraperitoneal injection. Finally, we recorded the LFP signals.

### 3.2. Experimental system

**Fig 1:**
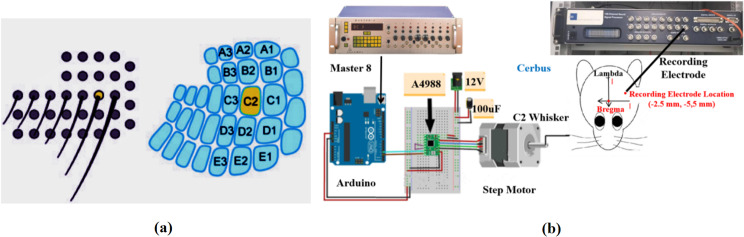
Whiskers and corresponding barrel columns and experimental system. (a) Whiskers and corresponding barrel columns: A schematic representation of the rat’s whisker system and the corresponding barrel columns in the primary somatosensory cortex (S1). The C2 whisker (yellow) is highlighted to indicate its role in locating the C2 barrel column. (b) Whisker stimulation system: The experimental setup for mechanical stimulation of the C2 whisker. The system includes an L-shaped tube, step motor, Arduino UNO R3 controller, A4988 motor driver, Master 8 pulse generator and Cerbus recording device.

Rats’ whiskers are sensitive detectors for tactile information, allowing them to navigate, recognize, and perceive their surroundings. As shown in Fig 1a, each whisker corresponds to a specialized barrel structure in the somatosensory cortex of the rat brain [1]. To capture specific neural signals triggered by whisker stimulation, this study applied mechanical stimulation to a single whisker. Fig 1b shows that an L-shaped tube is connected to the shaft of a stepper motor, and the target whisker (C2 of the large barrel column) is inserted into the tube. The Arduino UNO R3 controller and A4988 stepper motor driver together drive the motor shaft rotation, while a pulse generator (Master 8, A.M.P.I.) provides trigger signal. The Cerbus device is connected to recording electrodes to collect real-time LFP signals following whisker stimulation.

Referring to the previous studies by Tang et al. [25] and Tsytsarev et al. [26], in this study, the whisker was deflected counterclockwise from the head to the tail at a speed of 200 mm/s and then returned to its original position. Each deflection had an amplitude of 0.2 mm and employed a “ramp-and-hold” stimulus mode with a rise/fall time of 1 ms, maintaining a total deflection duration of 3 ms per stimulus. The interval between each stimulus was 1.5 seconds, and the entire experiment was repeated 200 times to ensure the reliability of cortical responses.

## 4. Experimental results

This section first present the barrel cortex LFP signals under different anesthetics protocols by whisker stimulation. Then we conduct a comparative analysis, emphasizing the robustness of the signals collected through our protocol. Finally, we demonstrate that our protocol results in minimal alterations in physiological parameters, highlighting its efficacy and safety.

### 4.1. The barrel cortex LFP signals under whisker stimulation with different anesthetic protocols

**Fig 2:**
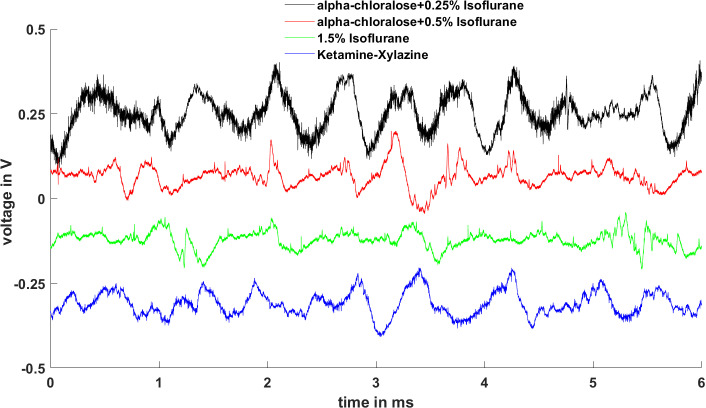
Comparison of EEG signals under different anesthetic protocols. Alpha-chloralose combined with 0.25% Isoflurane anesthesia is represented in black; Alpha-chloralose combined with 0.5% Isoflurane anesthesia is represented in red; 1.5% Isoflurane is represented in green; and Ketamine-Xylazine anesthesia is represented in blue.

**Fig 3:**
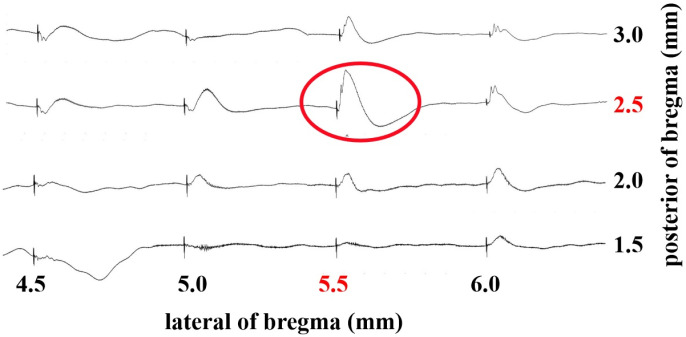
Precise localization of the C2 barrel column highlighted by red dashed circle in the somatosensory cortex.

The 30 animals were randomly assigned to three groups, with each group receiving one of the anesthetic protocols to ensure equal representation in the sample. We used AgCl extracellular electrode to record the animals’ brain EEG under alpha-chloralose, Isoflurane, and Ketamine-xylazine anesthetic protocols before whisker stimulation. When using a low concentration of Isoflurane (0.5%) or alpha-chloralose alone for anesthesia, animals typically wake within half an hour, displaying responses such as toe or ear twitches. Fig 2 showed stable EEG signals obtained under three anesthesia protocols. It can be seen that with alpha-chloralose combined with 0.25% Isoflurane, the EEG signals remain stable but include some muscle noise; with alpha-chloralose +  0.5% Isoflurane, the EEG signal improves significantly. Using 1.5% Isoflurane alone eliminates muscle noise in the EEG. Although Ketamine-Xylazine anesthesia has a stronger effect than 1.5% Isoflurane, it still introduces some muscle noise. These findings indicate that the combination of alpha-chloralose and 0.5% Isoflurane is an optimal approach for acquiring high-quality EEG signals from the animal brain. Accordingly, we employed this combination to record barrel cortex LFP signals in response to whisker stimulation. We stimulated the C2 whisker to find the precise C2 barrel column location. As shown in Fig 3, we can clear see that the C2 barrel column is located at 2.5 mm posterior and 5.5 mm lateral to bregma, within the left hemisphere.

**Fig 4:**
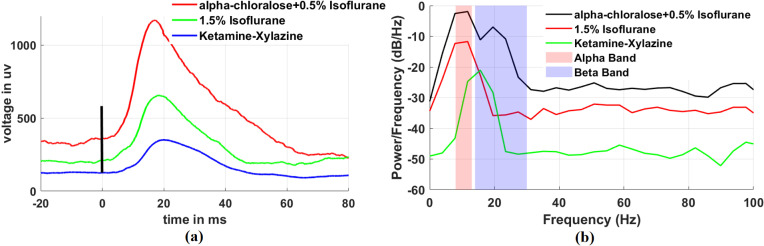
Comparison of barrel cortex LFP signals and corresponding power spectral density (PSD) across different anesthetic protocols. (a) Barrel cortex LFP signals recorded during whisker stimulation under three anesthetic conditions: alpha-chloralose +  0.5% Isoflurane (red), 1.5% Isoflurane (green), and Ketamine-Xylazine (blue). The time axis represents the duration of whisker stimulation in milliseconds, and the voltage axis represents the LFP signal amplitude in microvolts (µV). The peak LFP amplitude is highest with alpha-chloralose +  0.5% Isoflurane, followed by 1.5% Isoflurane and Ketamine-Xylazine. (b) Power spectral density (PSD) analysis of LFP signals across different anesthetic protocols. The alpha (8–13 Hz) and beta (13–30 Hz) frequency bands are highlighted with shaded regions (pink for alpha, blue for beta). The PSD comparison shows that alpha-chloralose +  0.5% Isoflurane significantly amplifies power in the alpha and beta frequency ranges, while 1.5% Isoflurane and Ketamine-Xylazine show reduced power in these bands.

We also analyzed the barrel cortex LFP signals and their corresponding Power Spectral Density (PSD) under whisker stimulation across different anesthetic protocols. As illustrated in Fig 4a, significant differences in the barrel cortex LFP signal amplitudes and peak latencies induced by whisker stimulation were observed under different anesthetic protocols. The peak amplitude under alpha-chloralose anesthesia (~800 µV) was approximately twice that under Isoflurane (~400 µV) and four times that under Ketamine-Xylazine (~200 µV). Furthermore, the peak latency of the LFP response under alpha-chloralose anesthesia was notably shorter (~17 ms) compared to Isoflurane (~18 ms) and Ketamine-Xylazine (~20 ms). These differences in both amplitude and latency likely reflect the distinct effects of each anesthetic protocol on cortical desynchronization, neural responsiveness, and signal transmission efficiency. Alpha-chloralose, by preserving excitability and minimizing inhibition, significantly enhances neural responsiveness and reduces latency, making it superior for capturing sensory-evoked LFP signals. In contrast, the stronger cortical suppression associated with Isoflurane and Ketamine-Xylazine not only results in lower LFP amplitudes but also prolongs peak latencies, indicating slower cortical response times. Fig 4b provides a detailed comparison of the PSD of LFP signal responses induced by whisker stimulation under three different anesthetic conditions. Compared to Isoflurane and Ketamine-Xylazine anesthesia, alpha-chloralose anesthesia significantly enhances the power of LFP signals in the low-frequency (alpha band, 8–13 Hz) and mid-frequency (beta band, 13–30 Hz) ranges. Notably, while delta band activity (0.5–4 Hz) typically dominates under deep anesthesia, it is markedly suppressed under alpha-chloralose anesthesia. This suppression may also be attributed to cortical desynchronization induced by whisker stimulation and the unique pharmacological properties of alpha-chloralose, which maintain neural responsiveness and amplify oscillatory activities in the alpha and beta bands. These findings not only highlight the superior ability of alpha-chloralose anesthesia to enhance the intensity and frequency response of neural signals induced by whisker stimulation but also reveal differences in the modulation of delta, alpha, and beta band activities across different anesthetic protocols. This modulation is of critical importance for optimizing neural signal acquisition in in vivo brain stimulation experiments and provides valuable insights for both research and clinical applications.

### 4.2. The barrel cortex LFP signal robustness

**Fig 5:**
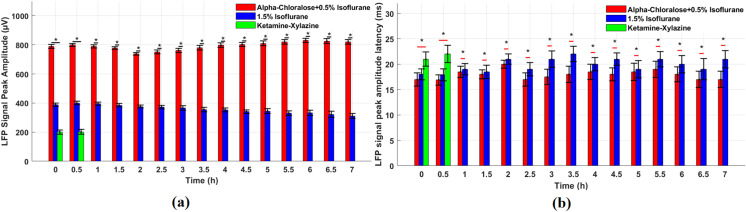
Longitudinal comparison of barrel cortex LFP signal amplitudes and latencies under different anesthesia protocols. (a) Mean and SEM of LFP signal amplitudes across 7 hours: This graph displays the mean and standard error of the mean (SEM) of the barrel cortex LFP signal amplitudes recorded under whisker stimulation for three anesthesia protocols: Alpha-chloralose +  0.5% Isoflurane (red), 1.5% Isoflurane (blue), and Ketamine-Xylazine (green). Alpha-chloralose consistently demonstrates the highest amplitude (~800 µV), significantly surpassing Isoflurane (~400 µV) and Ketamine-Xylazine (~200 µV) at all time points (*p < 0.05). *Represents the significance. (b) Mean and SEM of LFP signal peak latencies across 7 hours: This graph shows the mean and SEM of peak latencies of the LFP signals under whisker stimulation across three anesthesia protocols. Alpha-chloralose +  0.5% Isoflurane (red) demonstrates the shortest latency (~17 ms), compared to 1.5% Isoflurane (~20 ms) and Ketamine-Xylazine (~22 ms). Independent t-tests confirm statistically significant latency differences (*p < 0.05), particularly between Alpha-chloralose +  0.5% Isoflurane and 1.5% Isoflurane protocols. *Represents the significance.

We verified that the proposed anesthesia protocol demonstrates greater robustness in signal stability and anesthesia depth control. The experiment indicates that alpha-chloralose anesthesia reaches its deepest state at 2 hours, followed by a gradual recovery of neural activity and an enhanced response to whisker stimulation. Under whisker stimulation, we monitored the amplitude and peak latency of LFP signals in the barrel cortex to evaluate the effects of different anesthesia protocols. This evaluation method is similar to the one used in previous studies [27]. Fig 5 presents the mean and standard error of the mean (SEM) of LFP signal amplitudes and peak latencies under three anesthesia protocols. Compared to Isoflurane or Ketamine-Xylazine, alpha-chloralose anesthesia protocol has a greater LFP signal strength and a shorter latency period. Initially, the LFP signal amplitudes and peak latencies were 790 ± 13 uV and 17 ± 1.3 ms for alpha-chloralose, 388 ± 11 uV and 18 ± 1.1 ms for Isoflurane, and 201 ± 14 uV and 21 ± 1.4 ms for Ketamine-Xylazine. Then 800 ± 10 uV and 16.9 ± 1.0 ms, 400 ± 12 uV and 17.9 ± 1.2 ms, 202 ± 17 uV and 22 ± 1.7 ms after 0.5 hours, respectively. After 7 hours, they became to 820 ± 16 uV and 17 ± 1.6 ms, and 311 ± 17 uV and 21 ± 1.7 ms. Repeated measures ANOVA confirmed that the differences in LFP signal strength among the anesthesia protocols were statistically significant (*p < 0.05). Moreover, independent samples t-tests revealed a significant difference in peak latency between the alpha-chloralose +  0.5% Isoflurane and 1.5% Isoflurane protocols (*p < 0.05), indicating that the combination of alpha-chloralose and low-dose Isoflurane results in faster neural responses compared to higher concentrations of Isoflurane. In conclusion, the alpha-chloralose anesthesia protocol not only supports prolonged LFP signal recording in the barrel cortex but also significantly enhances the reliability of the data by maintaining high signal strength and reducing latency over time. This makes it a superior choice for long-term neural recordings compared to other commonly used anesthesia protocols such as Isoflurane and Ketamine-Xylazine.

### 4.3. Heart rate and body temperature changes under different anesthetic protocols

**Fig 6:**
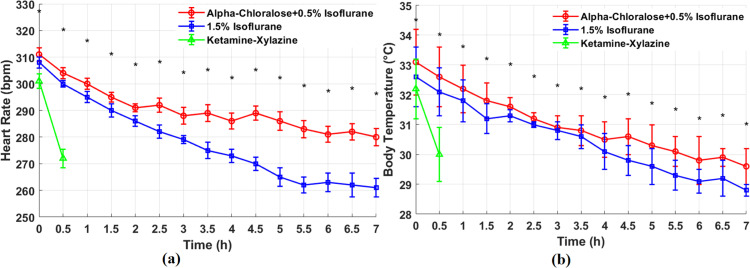
Comparative analysis of heart rate and body temperature changes over time under different anesthetic protocols. (a) Mean and SEM of heart rate changes over 7 hours for animals anesthetized with alpha-chloralose +  0.5% Isoflurane, 1.5% Isoflurane, and Ketamine-Xylazine. Statistical significance (*p < 0.05) was observed between alpha-chloralose and Isoflurane groups. *Represents the significance. (b) Mean and SEM of body temperature changes over 7 hours for animals anesthetized with alpha-chloralose +  0.5% Isoflurane, 1.5% Isoflurane, and Ketamine-Xylazine. Statistical significance (*p < 0.05) was noted between alpha-chloralose and Isoflurane groups. *Represents the significance.

As shown in Fig 6, we can clearly see the mean and SEM changes of heart rate and body temperature for 3 group animals under alpha-chloralose, Isoflurane, and Ketamine-Xylazine anesthetic protocols as anesthesia time going. The anesthetic effect of Ketamine-Xylazine lasts only 0.5 hours, and its potential lethal risk precludes repeated use on the same animal. Therefore, we recorded the physiological parameters for Ketamine-Xylazine over 0.5 hours, and for alpha-chloralose and Isoflurane over 7 hours.

Fig 6a clearly showed that at the beginning of the experiment, the baseline heart rates were 311 ± 2.6 beats per minute (bpm) for alpha-chloralose, 308 ± 3.1 bpm for Isoflurane, and 301 ± 2.8 bpm for Ketamine-Xylazine. 0.5 hours later, the heart rates decreased to 304 ± 2.0 bpm, 300 ± 1.2 bpm, and 272 ± 3.5 bpm, respectively. After 7 hours, the heart rates in the alpha-chloralose and Isoflurane groups further dropped to 280 ± 3.2 bpm and 261 ± 3.5 bpm, respectively. The results of one-way Analysis of Variance (ANOVA) showed that the difference in heart rate between alpha-chloralose and Isoflurane was statistically significant (*p < 0.05), indicating that these two anesthetic protocols had significantly different effects on heart rate control. An independent samples *t-test* further confirmed this difference (*p < 0.05), with the alpha-chloralose group showing a relatively stable decrease in heart rate, while the Isoflurane group exhibited a greater drop in heart rate. These findings suggest that alpha-chloralose can induce a relatively stable decrease in heart rate, making it particularly suitable for experiments involving long-term data collection. In contrast, Ketamine-Xylazine led to a rapid and pronounced decrease in heart rate, limiting its applicability for long-term observations.

Fig 6b present a typical temperature fluctuations among all experimental conditions. Initially, the baseline body temperature were 33.1 ± 1.3 °C for alpha-chloralose, 32.6 ± 1.8 °C for Isoflurane, and 32.2 ± 2.0 °C for Ketamine-Xylazine. Then decreased to 32.6 ± 2.0 °C, 32.1 ± 1.8 °C, and 31 ± 1.6 °C after 0.5 hours, respectively. After 7 hours, they became to 29.6 ± 1.2 °C, and 28.8 ± 2.2 °C. The results of a one-way ANOVA analysis indicated that the difference in body temperature fluctuations between alpha-chloralose and Isoflurane was statistically significant (*p < 0.05), suggesting that these two anesthetic protocols had significantly different effects on body temperature stability. An independent samples t-test further confirmed this difference (*p < 0.05), with the alpha-chloralose group showing a relatively stable decrease in body temperature over time, while the Isoflurane group exhibited a more pronounced drop in temperature. This suggests that alpha-chloralose can maintain a relatively stable decrease in body temperature, making it particularly suitable for long-term neural recording experiments where temperature stability is crucial. Stable temperature control is essential for accurate measurement of nervous system function, as excessively low or high body temperatures may alter neural signal conduction speed and neuronal activity, thereby affecting the reliability of LFP data. In contrast, Ketamine-Xylazine causes a rapid drop in body temperature, increasing the risk of data fluctuations and limiting its applicability for prolonged stable observations.

## 5. Discussion

The significant differences in the peak amplitudes and latencies of the barrel cortex LFP signals and heart rate and body temperature changes observed under different anesthetic protocols can be attributed to the distinct pharmacological properties of the anesthetics and their effects on neural excitability and cortical dynamics.

1) Alpha-chloralose’s superior signal enhancement and physiological stability: Alpha-chloralose anesthesia demonstrated significantly higher LFP peak amplitudes (~800 µV) and shorter peak latencies compared to Isoflurane and Ketamine-Xylazine. This superior performance can be attributed to alpha-chloralose’s unique ability to maintain a balance between excitatory and inhibitory neural networks [27,28]. By modulating GABAergic and glutamatergic pathways, alpha-chloralose preserves neuronal excitability and enhances cortical responsiveness to sensory stimulation [29,30]. Additionally, alpha-chloralose induces stable physiological conditions, including gradual and controlled reductions in heart rate and body temperature, minimizing fluctuations. This stability ensures a conducive environment for accurate and consistent neural recordings during prolonged experiments, further enhancing neural oscillations in the alpha and beta bands critical for sensory processing and sensorimotor integration.
2) Isoflurane’s cortical suppression and physiological instability: Isoflurane, a volatile anesthetic, acts as a GABA_A receptor agonist and NMDA receptor antagonist, inducing strong cortical suppression. While it effectively maintains anesthesia, Isoflurane significantly reduces neuronal excitability, resulting in diminished LFP amplitudes (~400 µV) and prolonged peak latencies. Moreover, Isoflurane causes more pronounced declines in physiological parameters such as heart rate and body temperature, further contributing to reduced neural responsiveness. This dual effect of cortical suppression and physiological instability limits Isoflurane’s suitability for long-term neural recording [31–33].
3) Ketamine-Xylazine’s limitations on signal and physiology: Ketamine-Xylazine anesthesia resulted in the lowest LFP amplitudes (~200 µ V) and the longest peak latencies among the tested protocols. Ketamine, an NMDA receptor antagonist, suppresses excitatory synaptic transmission, while Xylazine, an α2-adrenergic receptor agonist, induces deep sedation and muscle relaxation. However, this combination often causes rapid and severe reductions in heart rate and body temperature [34,35]. These physiological disruptions impair cortical function and neural signal transmission, leading to attenuated LFP responses and limiting Ketamine-Xylazine’s applicability in long-term or sensory-evoked neural studies [36].
4) Mechanistic insights into enhanced signal amplitudes: The observed differences in LFP signal amplitudes and latencies are closely linked to the degree of cortical desynchronization and physiological stability induced by each anesthetic. Alpha-chloralose promotes moderate cortical desynchronization and maintains stable physiological parameters, allowing for enhanced neural responses to whisker stimulation. In contrast, Isoflurane and Ketamine-Xylazine induce stronger cortical suppression and greater physiological fluctuations, reducing the frequency range and amplitude of neural responses. Alpha-chloralose’s mild central nervous system suppression minimizes its impact on brain metabolism and blood flow, providing an optimized environment for robust sensory-evoked potentials while ensuring consistent heart rate and body temperature.

The barrel cortex LFP signals and their corresponding PSD under whisker stimulation underscore the importance of choosing appropriate anesthetic protocols to optimize neural signal acquisition in sensory-evoked studies. Alpha-chloralose’s ability to preserve neural excitability and enhance cortical responses makes it a superior choice for experiments requiring precise and stable LFP recordings. Meanwhile, the observed dominance of alpha and beta bands in the power spectrum, rather than typical delta oscillations under anesthesia, can be attributed to the experimental paradigm and the choice of anesthetic agent. Whisker stimulation enhances cortical oscillations in the alpha and beta bands, associated with sensory processing and sensorimotor integration [33,36]. Alpha-chloralose anesthesia preserves cortical responsiveness, amplifying alpha and beta activities while suppressing delta oscillations typical of deep anesthesia [30,37]. These results provide valuable insights for future studies aiming to refine anesthetic techniques for in vivo neural signal acquisition.

In the course of anesthesia, the gradual decrease in body temperature and heart rate over time can primarily be attributed to the effects of anesthetics, such as Isoflurane and alpha-chloralose, which act as central nervous system depressants inhibiting sympathetic nerve activity [38]. Isoflurane, in particular, induces rapid vasodilation, leading to significant heat loss and a rapid drop in body temperature [39]. In contrast, alpha-chloralose demonstrates a more gradual and stable decline in body temperature and heart rate, potentially due to its milder impact on the hypothalamic thermoregulation center [40]. Furthermore, alpha-chloralose offers a longer duration of anesthetic effects (up to 7 hours) compared to Ketamine-Xylazine, which lasts only about 30 minutes, making it more suitable for prolonged experiments [41]. In our study, while both heart rate and body temperature decreased under alpha-chloralose anesthesia, the rate of change was more stable than with Isoflurane, allowing for better maintenance of physiological stability during long-term neural recordings.

## 5. Conclusions

This study proposes an anesthesia protocol combining Isoflurane, Dexdomitor, and alpha-chloralose to optimize the quality of barrel cortex LFP signals elicited by whisker stimulation. It systematically compares different anesthesia protocols in terms of signal characteristics, physiological stability, and neural response latency. The novelty of this study is highlighted in three key aspects: 1) In-depth Exploration of the Anesthesia Protocol and Signal Enhancement Mechanism: The experimental results demonstrate that alpha-chloralose anesthesia significantly enhances the amplitude of barrel cortex LFP signals (approximately twice that of Isoflurane and four times that of Ketamine-Xylazine) and shortens peak latency. Compared to conventional anesthesia methods, these advantages are attributed to the desynchronization and balancing mechanisms facilitated by alpha-chloralose through the modulation of GABA and glutamate pathways. This mechanism not only improves cortical responsiveness to whisker stimulation but also avoids the excessive suppression of neuronal excitability seen with Isoflurane and the physiological fluctuations induced by Ketamine-Xylazine. 2) Explanation of the Mechanisms Underlying Anesthesia-Induced Differences: The differences in LFP signal characteristics across anesthesia protocols can be attributed to their distinct pharmacological actions: Isoflurane induces deep neuronal inhibition by activating GABA_A receptors and inhibiting NMDA receptors, leading to reduced LFP signal amplitude and delayed responses. Ketamine-Xylazine causes significant physiological fluctuations (e.g., heart rate and body temperature decreases) due to NMDA receptor blockade and α2-adrenergic agonist effects, which increase signal variability. Alpha-chloralose maintains a dynamic balance between excitatory and inhibitory neural networks, enabling the cortex to respond to whisker stimulation rapidly and efficiently while reducing delta-band synchronized slow-wave activity. Furthermore, alpha-chloralose’s mild central nervous system suppression makes it suitable for long-term stable recordings. 3) Practical Methodological Value: The study validates the reliability and advantages of alpha-chloralose anesthesia for long-term neural recordings, evidenced by sustained high signal strength (over 800 µ V for 7 hours) and relatively stable heart rate and body temperature. In contrast, Isoflurane caused more pronounced physiological parameter fluctuations, while Ketamine-Xylazine’s rapid induction of heart rate and body temperature drops limited its applicability for prolonged experiments. These findings provide technical guidance for optimizing neural signal acquisition, particularly in sensory neuroscience research that demands high sensitivity and minimal interference. Through a detailed exploration of anesthesia mechanisms and signal differences, this study lays a solid foundation for further advancements in the field of sensory neuroscience.

## References

[1] Petersen CC. The functional organization of the barrel cortex. Neuron. 2007;56(2):339–55. doi: 10.1016/j.neuron.2007.09.017 17964250

[2] Feldmeyer D. Excitatory neuronal connectivity in the barrel cortex. Front Neuroanat. 2012;6(24):1–22. doi: 10.3389/fnana.2012.00024 22798946 PMC3394394

[3] Schubert D, Staiger JF, Cho N, Kotter R, Zilles K, Luhmann HJ. Layer-specific intracolumnar and transcolumnar functional connectivity of layer V pyramidal in rat barrel cortex. J Neurosci. 2001;21(10):3580–92. doi: 10.1523/JNEUROSCI.21-10-03580.2001 11331387 PMC6762473

[4] Fox K. Barrel Cortex. Cambridge: Cambridge University Press; 2008.

[5] Herreras O. Local field potentials: myths and misunderstandings. Front Neural Circuits. 2016;10(101):1–16. doi: 10.3389/fncir.2016.00101 28018180 PMC5156830

[6] Kajikawa Y, Schroeder CE. How local is the local field potential recordings. Neuron. 2011;72(5):847–58. doi: 10.1016/j.neuron.2011.09.029 22153379 PMC3240862

[7] Franks NP, Lieb WR. Mechanisms of general anesthesia. Environ Health Perspect. 1990;87:199–205. doi: 10.1289/ehp.9087199 2269226 PMC1567828

[8] Hemmings HC, Akabas MH, Goldstein PA, Trudell JR, Orser BA, Harrison NL. Emerging molecular mechanisms of general anesthetic action. Trends Pharmacol Sci. 2005;26(10):503–10. doi: 10.1016/j.tips.2005.08.006 16126282

[9] White PF. Clinical uses of Intravenous Anesthetic and Analgesic Infusions. Anesth Analg. 1989;68(2):161–71. doi: 10.1213/00000539-198902000-00017 2643889

[10] Eger EI. The pharmacology of inhaled anesthetics. Semin Anesth Perioperat Med Pain. 2005;24(2):89–100. doi: 10.1053/j.sane.2005.04.004

[11] Zanos P, Gould TD. Mechanisms of ketamine action as an antidepressant. Mol Psychiatry. 2018;23(4):801–11. doi: 10.1038/mp.2017.255 29532791 PMC5999402

[12] Kaloptia K, Armakolas A, Pjilippou A, Zarros A, Angelogianni P. Ketamine-induced neurotoxicity in neurodevelopment: a synopsis of main pathways based on recent in vivo experimental findings. J Anaesthesiol Clin Pharmacol. 2021;37(1):37–42. 10.4103/joacp.JOACP_415_19 34103820 PMC8174420

[13] Lin D, Zuo Z. Isoflurane induces hippocampal cell injury and cognitive impairment impairments in adult rats. Neuropharmacology. 2011;61(8):1354–9. doi: 10.1016/j.neuropharm.2011.08.011 21864548 PMC3189329

[14] Michaels JE, Barnes CA, Meltzer J. Effects of isoflurane on physiological parameters in murine subcutaneous tumor allografts measured via diffuse reflectance spectroscopy. Biomed Opt Express. 2018;9(6):2871–86. 10.1364/BOE.9.002871 30258696 PMC6154201

[15] Sang SO, John MH, Catrina SR, Kelli AS, Eva LF. The effects of anesthetics on measures of nerve conduction velocity in male C57Bl6/J mice. Neurosci Lett. 2010;483(2):127–31. 10.1016/j.neulet.2010.07.076 20691755 PMC2941214

[16] Naaz S, Ozair E. Dexmedetomidine in current anesthesia practice. J Clin Diagn Res. 2014;8(10):GE01–4. doi: 10.7860/JCDR/2014/9624.4946 25478365 PMC4253183

[17] Sloan TB. Anesthetic Effects on electrophysiologic recordings. J Clin Neurophysiol. 1998;15(3):217–26. doi: 10.1097/00004691-199805000-00005 9681559

[18] Matthew RI, Carlos MC, Shinbe C, Eugene AK. Basic physiological effects of ketamine-xylazine mixture as a general anesthetic preparation for rodent surgeries. Brain Resear. 2023;1804(148251):1–12. 10.1016/j.brainres.2023.148251 PMC9975069 36690168

[19] An S, Yang JW, Sun H, Kilb W, Luhmann H. Long-term potentiation in the neonatal rat barrel cortex in vivo. J Neurophysiol. 2012;32(28):9811–6. 10.1523/JNEUROSCI.1212-12.2012 PMC6622258 22787036

[20] Yuan Y, Li S, Wu L, Wang J. The efficient method to get better raw brain signal on rat anesthetics experiment. J Pharmacol Toxicol Methods. 2024;129(107551):1–8. 10.1016/j.vascn.2024.107551 39245416

[21] Pani P, Bello FD, Brunamonti E, Valeria DA, Odysseas P, et al. Alpha and beta band oscillations subserve different processes in reactive control of limb movements. Front Behav Neurosic. 2014;5(8):383. 10.3389/fnbeh.2014.00383 PMC4220745 25414649

[22] Luckl J, Keating J, Greenberg H. Alpha-chloralose is a suitable anesthetic for chronic focal cerebral ischemia studies in the rat: a comparative study. Brain Res. 2007;1191::157–67. 10.1016/j.brainres.2007.11.037 PMC2266075 18096143

[23] Redwan SM, Uddin MP, Ulhaq A, Sharif MI, Krishnamoorthy G. Power spectral density based resting-state EEG classification of first-episode psychosis. Scientif Rep. 2024;14(15154):1–12. 10.1038/s41598-024-66110-0 PMC11219808 38956297

[24] White WJ, Field KJ. Anaesthesia and surgery of laboratory animals. Vet Clin N Am Small Anim Pract. 1987;17(5):989–1017. doi: 10.1016/s0195-5616(87)50102-4 3310373

[25] Tang Q, Tsytsarev V, Yan F, Wang C, Erzurumlu RS, Chen Y. In vivo voltage-sensitive dye imaging of mouse cortical activity with mesoscopic optical tomography. Neurophotonics. 2020;7(4):041402. doi: 10.1117/1.NPh.7.4.041402 33274250 PMC7708784

[26] Tsytsarev V, Pope D, Pumbo E, Garver W. Intrinsic optical imaging of directional selectivity in rat barrel cortex: application of a multidirectional magnetic whisker stimulator. J Neurosci Methods. 2010;189(1):80–3. doi: 10.1016/j.jneumeth.2010.03.010 20304008

[27] Austin VC, Blamire AM, Allers KA, Sharp T, Styles P, Matthews PM, et al. Confounding effects of anesthesia on functional activation in rodent brain: a study of halothane and alpha-chloralose anesthesia. Neuroimage. 2005;24(1):92–100. doi: 10.1016/j.neuroimage.2004.08.011 15588600

[28] Peng SL, Chiu H, Wu CY, Huang CW, Chung. The effects of caffeine on cerebral metabolism during alpha-chloralose anesthesia differs from isoflurane anesthesia in rats brain. J Psychophar. 2019;236(6):1749–57. 10.1007/s00213-018-5157-4 30604185

[29] Ueki M, Mies G, Hossmann KA. Effects of alpha-chloralose, halothane, pentobarbital and nitrous oxide anesthesia on metabolic coupling in somatosensory cortex of rats. Acta anesthesia Scand. 1992;36(4):318–22. 10.1111/j.1399-6576.1992.tb03474.x 1595336

[30] Wang Y, Li X, Chen Z. Effects of anesthetics on sensory-evoked potentials. Neurophysiol Clin. 1993;23(3):141–62. 10.1016/s0987-7053(05)80227-8 8326927

[31] Antkowiak B. How do general anaesthetics work? Naturwissenschaften. 2002;88(5):201–13. doi: 10.1007/s001140100230 11482433

[32] Rudolph U, Antkowiak B. Molecular and neuronal substrates for general anaesthetics. Nat Rev Neurosci. 2004;5(9):709–20. doi: 10.1038/nrn1496 15322529

[33] Land R, EngLer G, Kral A, Engel A. Auditory evoked bursts in mouse visual cortex during isoflurane anesthesia. PLoS One. 2012;7(11):49855. doi: 10.1371/journal.pone.0049855 PMC3504086 23185462

[34] Franks NP. General anaesthesia: from molecular targets to neuronal pathways of sleep and arousal. Nat Rev Neurosci. 2008;9(5):370–86. doi: 10.1038/nrn2372 18425091

[35] Virtanen R. Pharmacological profiles of medetomidine and its antagonist, atipamezole. Acta Vet Scand Suppl. 1989;85:29–37. 2571275

[36] Lee H, Tanabe S, Wang S, Hudetz AG. Different effect of anesthesia on visual cortex neurons with diverse population coupling. Neurosci. 2021;485:108–19. doi: 10.1016/j.neuroscience.2020.11.043 PMC7925367 33309966

[37] Haslinger R, Ulbert I, Moore CI, Brown EN, Devor A. Analysis of LFP phase predicts sensory response of barrel cortex. J Neurophysiol. 2006;96(3):1658–63. . doi: 10.1152/jn.01288.2005 16775200

[38] Khan ZH, Arab S, Emami B. Comparative of the effects of anesthesia with isoflurane and total intravenous anesthesia on the intensity of body temperature reduction anesthesia and incidence of postoperative chills. Acta Medical Iranica. 2011;49(7):425–32. 21960073

[39] Yang CF, Chen MY, Chen TI, Cheng CF. Dose-dependent effects of isoflurane on cardiovascular function in rats. Tzu Chi Medical Journal. 2014;26(3):119–22. doi: 10.1016/j.tcmj.2014.07.005

[40] Low LA, Bauer LC, Klaunberg BA. Comparing the Effects of alpha-chloralose and isoflurane upon mouse physiology. PLoS One. 2016;11(5):e0154936. doi: 10.1371/journal.pone.0154936 27148970 PMC4858227

[41] Luckl J, Keating J, Greenberg JH. Alpha-chloralose is a suitable anesthetic for chronic focal cerebral ischemia studies in the rat: a comparative study. Brain Res. 2008;1191:157–67. doi: 10.1016/j.brainres.2007.11.037 18096143 PMC2266075

